# Michigan Dental Association

**Published:** 1867-08

**Authors:** G. Leary


### Proceedings of Societies.

### PROCEEDINGS OF THE MICHIGAN DENTAL ASSOCIATION.

BY G. LEARY.

THE twelfth annual meeting of this Association was held at Adrian, Jan. 22, Dr. G. L. Field, of Detroit, presiding. The various sessions were well attended, and its deliberations throughout were characterized by earnestness and vigorous industry.

After the reading of the Constitution and election of members, the reports of committees were called for. The Committee appointed to confer with the Regents of the State University with reference to the establishment of a Dental Chair in that institution reported favorable progress, and by its own request was discharged.

The Society next proceeded to the election of officers for the ensuing year, with the following result:

*President*—Dr. J. A. Harris.

*Vice-President*—Dr. B. Bannister.

*Secretary*—Dr. G. Leary.

*Treasurer*—Dr. H. Benedict.

At the evening session, Drs. Watling and Benedict were appointed a Committee to conduct the President elect to the chair. On assuming its duties he thanked the Association for the honor conferred, and solicited the indulgence and co-operation of all in the discharge of the duties of his office.

President Field, on retiring delivered a stirring valedictory address, which was received with evident interest. A University Committee was again appointed to urge the establishment of one or more Dental Professorships.

On motion of Dr. Watling, a committee of five was appointed to draft a paper for consideration, petitioning the Legislature to legalize the practice of Dentistry. The report was accepted and adopted, and the committee was further instructed to procure printed copies and distribute among the members of the profession to secure signatures.

### SECOND DAY—MORNING SESSION.

After the transaction of business, the subjects for discussion were announced.

1st. Preservation of the Temporary Teeth.

Dr. G. W. Stone, in opening the discussion said, that he regards the subject as one deserving special attention. The retention of the deciduous teeth is of such vital importance in securing a well formed and healthy permanent set, that they are worthy of more attention than is generally given them. He finds them quite susceptible to treatment, and therefore deprecates the practice of many in extracting these organs by wholesale simply because of pain. He counsels nice discrimination in their treatment, and a very abstemious use of the forceps.

Dr. Field coincides with the views expressed in regard to retaining the temporary teeth, but cannot think them so generally sacrificed as Dr. S. fears. He urges the employment of all means, medical and mechanical for their preservation, and believes that with judicious treatment they may be kept the proper time.

Dr. Owen uses every appliance for retaining the temporary teeth that are not hopelessly involved in disease. The same sentiments were expressed by Drs. Holmes, Rix and Knapp.

Dr. Field believes that the diet of children is generally ill chosen for securing a good temporary or permanent denture. Our careful abstraction of the bran from grains not only renders the flour less digestible, but also largely deprives it of its bone-making principle. He therefore advises the use of unbolted flôur and a plain nutritious diet generally.

Dr. Johnston indorses Dr. Field's views, and charges remissness of duty upon the profession in failing to properly instruct society as to the importance of retaining the deciduous teeth until naturally displaced.

Dr. Benedict related cases in practice, showing a gratifying success in the adoption of a plain wholesome diet and uniform habits of living. With the preceding speakers he disapproves the extraction of temporary teeth where it is possible to retain them in a comfortable condition.

Dr. Douglas advises entire abstinence from the use of fluids while eating. The salivary glands are thereby stimulated to a thorough performance of their functions, and thus the food is more properly prepared for the further process of digestion.

He believes that soda should be eschewed in the preparation of food, as it possesses strong affinity for the acids of the stomach, and when brought in contact with them, either neutralizes them or at least impairs their efficiency. He thinks that the observance of well chosen dietary rules and the prompt and thorough treatment of colds and other affections of the air passages with those of the alimentary canal, would greatly conduce to ward off, or at least mitigate the diseases to which the teeth are liable.

Dr. Field said, that neither children nor adults are sufficiently diligent in the use of the tooth-brush. His manner of using it is to apply to the margin of the gum and brush the tooth longitudinally instead of transversely as is the general practice.

Dr. Holmes would not undervalue the tooth-brush, but thinks the quill toothpick of greater importance, as by its use the interstices between the teeth can be freed from extraneous matter which the brush cannot reach.

Dr. Johnston prefers the dogwood toothpick to all others, on account of its antiseptic properties.

Dr. Stone said he advocates the use of the brush and toothpick also; but as these are simply conservatory appliances, they cannot affect the constitution of the tooth which is generally defective. The deciduous teeth nearly complete their formation during fœtal life, therefore if we would improve them in structure it must be done by treating the mother for this purpose during gestation.

Dr. Cahoon did not doubt the efficacy of such treatment when judiciously administered, but owing to feelings of delicacy on the part of such patients, did not think it capable of general adoption.

Dr. Harroun has had gratifying success in administering the salts of lime as a constitutional remedy for the teeth during intra-uterine life. He believes that generally the intelligent Dentist will not find any insurmountable obstacle to oppose the plan of treatment he may prescribe, and thinks if his patrons have confidence in his intelligence and integrity, he will experience little difficulty in securing their co-operation.

Dr. Holmes here called attention to the importance of elevating the standard of professional character and urged upon all the necessity of high attainments if they would bless mankind and adorn their profession.

Dr. Cahoon then called attention to the tendency of certain teeth to decay, prior to others in the same mouth. He has found the superiors generally the first to yield, and believes that the cause is attributable to mechanical violence inflicted by their antagonists.

Dr. Benedict has found that the inferior molars fail simultaneously with or prior to their antagonists, and believes that the cause is found in the extremes of temperature to which they are subjected in taking food and drink.

Dr. Finch has observed that the superior centrals first decay, then the inferior molars, and with Dr. B. regards the extremes of temperature the cause.

### AFTERNOON SESSION.

SUBJECT—Absorption of the Alveolar Process—Causes and Treatment.

Dr. Field related cases in practice which were successfully treated, by the use of iodine and creosote, alternated with glycerine as a palliative in the last stages of the cure.

Dr. Harroun has used the phenate of soda with much success in the treatment of the above disease, and has often noticed the beneficial effects of the iodide of potassium as a constitutional remedy.

Dr. Owen mentioned cases of recession of the gum and absorption of the process, where the general health was good, the digestive and assimilative functions unimpaired, and no accumulations of tartar could be discovered on the teeth. He regards advanced cases of this disease as being very difficult of successful treatment, and believes that when it is firmly established, it usually accomplishes the destruction of the teeth.

After some further remarks by various members, the subject was passed, and—

The Constitutional Effects of Diseased Teeth and Fangs were considered.

A general and interesting narration of cases in practice here occurred, showing the alarming nature which diseases of the teeth are liable to assume, and demonstrated the necessity of an intelligent and discriminating diagnosis in their treatment.

The attention of the Association was next occupied by a consideration of Hard Rubber and the Cummings Patent.

After thoroughly canvassing the matter the Society unanimously resolved to co-operate with Dental Associations to resist all encroachments upon the just rights of the profession.

On motion, It was ordered, that Thursday morning session be devoted to operations on the natural teeth.

### EVENING SESSION.

Association was called to order at 7.30 P. M. Dr. Benedict in the chair. Dr. Harroun was then requested to communicate any information he might have to offer concerning his mode of treating Cleft Palate.

He responded by saying that where the palate is entirely cleft, the operator must depend generally upon mechanical appliances alone. He has in such cases encountered great difficulty in obtaining a correct impression of the soft palate, on account of the great sensitiveness of this organ to the presence of foreign bodies. Yet in a good subject this difficulty may be overcome by manipulating the parts with the fingers, and with instruments. When the palate becomes tolerant of touch, he proceeds to take the impression immediately. The appliance usually consists of a body of hard rubber, with a velum of flexible rubber attached. Not having models and instruments at hand, he did not explain in detail the process of construction; but closed by stating that in some cases he had been so far successful, as to almost entirely remedy the defect in speech.

The regular order of discussion was then suspended, and the Association engaged in a mutual interchange of modes of operation pursued by each in the department of Mechanical Dentistry.

Dr. Holmes related in detail his method of remounting artificial teeth without changing their present articulation. It is as follows:

After obtaining the model, place the denture to be remounted in proper position thereon, and set the whole in the flask in the usual manner. Separate the flask, then heat the part containing the plate until the rubber is sufficiently softened, so that it may be removed without injury to the teeth or rivets; then remove the plate, and the case is ready for packing.

Dr. Benedict oils the plate, and heats slowly over a flame; picks off the teeth, and rearranges them on a new trial plate; then places the whole in the mouth, and secures the exact articulation.

Dr. B. here stated that he had received a circular from Dr. Hause, by which he learned that for a certain sum he could become acquainted with the method of remounting teeth as practiced by Dr. H. He said that it had always given him pleasure to place at the disposal of the profession every new idea and improvement of which he was possessed, that in turn he had sought by all proper means to avail himself of the researches of others; but he had not bought nor would he buy professional secrets. He considered the proposal of Dr. Hause as derogatory to professional character, and entirely at variance with the usages of the Association.

Dr. Hause in reply made the *amende* honorable, by declaring that at the time he did not regard such conduct exceptional. He hoped the Association would overlook the mistake, and promised that now and hereafter his discoveries should be free and for the benefit of all. He then gave in detail his mode of procedure, which did not differ essentially from that of Dr. Holmes.

The subject of Exposed Nerves was then taken up.

Dr. Stone said he regards extirpation and thorough filling of the cavity, the most efficient treatment for exposed nerve. He applies cobalt as a devitalizer, and fills immediately. By this process he believes that periosteal inflammation is avoided.

Drs. Benedict and Watling apply the arsenical paste, and defer filling until all danger of periostitis is past. After some further remarks the subject was dismissed, and the Association adjourned until 9 A. M. of Thursday. Pending the motion to adjourn, Dr. Owen tendered the compliments of the Dentists of Adrian, by inviting all present to a collation at the Lawrence House, immediately after adjournment. The invitation was cordially accepted.

### THIRD DAY—MORNING SESSION.

Association met pursuant to adjournment. The President in the chair.

Operations on the natural teeth was then announced as the order of business. After witnessing several operations in filling, in which were exhibited the merits of the various automatic instruments, the Association adjourned till 2.30 P. M.

### AFTERNOON SESSION.

The meeting was called to order by the President.

By request, the Committee on Automatic Mallets was permitted to defer its report until the next annual meeting.

On motion, Drs. J. Douglas, Finch, Knapp, Holmes, Bannister, Leary and Owen, were appointed delegates to the American Dental Association.

It was moved by Dr. Stone, that a committee of three be appointed to draft resolutions of respect and condolence in memory of Dr. D. A. Wilder, deceased. A committee was appointed, and reported the following, which was adopted:

WHEREAS this Association learns with grief and heartfelt sorrow of the untimely death of our much esteemed co-worker, Dr. D. A. Wilder. Therefore,

*Resolved*, That this Association has lost an active and efficient member, and the citizens of Jackson a valuable citizen and an accomplished Dentist.

*Resolved*, That we sympathize with the widow of the deceased in her bereavement, and tender her our heartfelt condolence.

On motion of Dr. Stone, the thanks of the Society were tendered to Dr. Finch for the use of his office, and to the Dentists of Adrian for their gentlemanly and courteous bearing toward the members of the Association.

The Association then adjourned to meet in Detroit, on the second Tuesday in October next.

